# Breast cancer in moroccan young women: a retrospective study

**DOI:** 10.1186/1756-0500-3-286

**Published:** 2010-11-08

**Authors:** Halima Abahssain, Issam Lalya, Fatima zahra EL M'Rabet, Nabil Ismaili, Rachid Razine, Mohammed Adnane Tazi, Hind M'rabti, Omar El Mesbahi, Nourddine Benjaafar, Redouane Abouqal, Hassan Errihani

**Affiliations:** 1 Department of medical oncology, National Institute of Oncology, Rabat, Morocco; 2 Department of radiotherapy, National Institute of Oncology, Rabat, Morocco; 3 Department of Medical Oncology, Hassan II University Hospital, Fez, Morocco; 4 Laboratory of Biostatistics, Epidemiology and Clinical Research, Rabat, Morocco; 5 Epidemiology Unit, National institute of oncology, Rabat, Morocco

## Abstract

**Background:**

Breast cancer is uncommon in young women and induces more aggressive biologic characteristics. Survival in young women has been widely studied in developed countries. Less favorable prognosis and low survival were found.

In Morocco, this study is the first investigation of clinical features, treatment and prognosis associated with breast cancer in young women.

**Findings:**

Four hundred and nine women aged 35 years or less were included in this study. All these women were diagnosed as having breast cancer at the National Institute of Oncology in Rabat, Morocco between 2003 and 2007. The relation between clinical and therapeutic characteristics and event-free survival (EFS) and overall survival (OS) were assessed by Cox regression analysis.

The median age of the patients was 32 years. Fifty three patients (13%) have metastatic disease at diagnosis and 356 patients (87%) had localised disease. In 57.9% of the cases, the estrogen receptors status was positive. The median follow-up was 32.2 months. After 3 years the survival rate was 80.6%. In the case of localised disease, OS and EFS at 3 years were 83.2% and 62.5%, respectively. OS and EFS at 3 years was higher in patients with stage I than patients with stage II and stage III (p = 0.001). Positive estrogen receptors was significantly associated to OS and EFS at 3 years compared to negative estrogen receptors (p = 0.001). Adjuvant chemotherapy, adjuvant radiotherapy and adjuvant hormone therapy were associated with net benefit in OS and EFS at 3 years. Cox regression analysis showed that negative ER was significantly associated with poorer OS (HR = 2.42, 95% CI = 1.25 - 4.66, p < 0.009) and poorer EFS (HR = 1.73, 95%CI = 1.05 - 2.86, p = 0.03). Stage III disease were associated to poorer EFS (HR = 5.35, 95%CI = 1.60 -17.84, p = 0.006).

**Conclusions:**

In Morocco, young women with breast cancer had less favorable prognosis. Multivariate analysis showed that negative hormone receptor status was associated with lower EFS and OS. Clinical trials should be launched to improve the survival of these young women with breast cancer.

## Background

Breast cancer in young women is uncommon. Approximately 2% of patients with breast carcinoma are age ≤ 35 years old at the time of diagnosis [1,2]. Information is limited in this category of women. The definition of "young women" varied from 30, 35, 40, 45, or even 50 years [3]. Young patients with breast cancer had more aggressive clinical and biological characteristics, less favorable outcome, and the disease was more linked to a genetic predisposition compared with the disease in older patients [4-7].

According to the 1998 St Gallen guidelines, age ≤ 35 was a poor prognostic factor and supported the use of more aggressive systemic therapy, including chemotherapy in all younger patients regardless of other factors [8].

In addition, other particularities must be studied in this category of patients: fertility, menopause induced by treatment, self-image and sexuality.

The aim of the present study was to investigate the epidemiological, clinical and treatment characteristics in young patients with breast cancer, and their relationship with event free survival and overall survival.

## Patients and methods

### Clinical data

The National Institute of Oncology database was used to identify patients with breast cancer aged 35 years or less at the time of diagnosis between 2003 and 2007. We excluded from the study patients who had not follow up after initial diagnosis. The scientific comity of National Institute of Oncology approved the retrospective review of the medical records for the purposes of the current study. Breast carcinoma diagnosis was made by biopsy of the breast tumor. Tumor staging was carried out according to the TNM classification 2002 modified in 2003. Histological tumor grading was performed using the Scarff Bloom and Richardson (SBR) histological system.

Immunohistochemical analysis to determine estrogen (ER) and progesterone receptor (PR) status was performed using standard procedures on 4-μm sections of paraffinembedded tissue specimens stained with the monoclonal antibodies 6F11 and 1A6 for ER and PR, respectively. Nuclear staining 10% was considered a positive result.

Patients were considered HER2-positive if they had immunohistochemistry (IHC) 3+ by DAKO HercepTest. Fluorescence in situ hybridization was used when IHC was 2+ and it was considered positive if we have ≥ two-fold amplification; confirmed by central testing. The hercept test was realized in the institute from 2004.

### Treatment

Patients how had metastatic disease at diagnosis received chemotherapy or hormonotherapy based on the characteristics of the tumors and the aggressiveness of the disease. This patients can receive palliative radiotherapy if indication. Patients with local disease had received corresponding local treatments (surgery plus radiotherapy) and systemic treatments (mainly adjuvant and/or neoadjuvante chemotherapy and endocrine therapy). The main surgical operations included radical mastectomy (Patey type mastectomy) and breast conserving surgery when permitted by tumor size according to the judgment of the multidisciplinary care team.

In our institute, adjuvant chemotherapy is indicated in the case of tumor size greater than or equal to 2 cm, positive nodal status, grade 2 or 3 SBR, amplification of HER 2 and age ≤ 35 years. Neoadjuvante chemotherapy was giving in patients with inflammatory or locally advanced breast cancer. For the patients who did not receive adjuvant chemotherapy, they have received neoadjuvante chemotherapy or they did not return after surgery for personal reasons.

For patients receiving neoadjuvante chemotherapy, a pathologic complete response was defined as no evidence of invasive carcinoma in the breast and the axillary lymph nodes at the time of surgery. Chevalier classification was used to classify histological response to neoadjuvante chemotherapy in the breast [9].

Anthracycline containing regimens were mainly used for adjuvant and/or neoadjuvante chemotherapy (FEC 100 protocol with Fluorouracil 500 mg/m^2^, Epirubicin 100 mg/m^2 ^IV, Cyclophosphamide 500 mg/m^2 ^and AC60 protocol with Doxorubicin 60 mg/m^2^, Cyclophosphamide 600 mg/m^2^) while CMF regimen (Cyclophosphamide 600 mg/m^2^, Metotrexate 40 mg/m^2^, Fluorouracil: 500 mg/m^2^) was administrated instead in some minor cases. Docetaxel was administered at a dose of 100 mg/m^2 ^when used in monotherapy and at a dose of 75 mg/m^2 ^in combination with anthracyclin. The choice of chemotherapy protocols depended on the availability of products at the time of the indication.

Patients with hormone receptor positive tumor specimens received tamoxifen at a dose of 20 mg daily for 5 years. Adjuvant radiotherapy was indicated the case of tumor size greater than 5 cm, invasion of the pectoral fascia, more than four metastatic axillary lymph nodes, positive surgical margin and breast conservation.

### Follow up

Patients were followed up until January 2010. All patients who are not reviewed in the last consultation were contacted again by telephone. Locoregional recurrence meant the recurrence in ipsilateral mammary glands, chest wall, or regional lymph nodes identified clinically or histologically, while distant metastasis referred to the metastatic carcinoma detected by clinical examination or imaging. Event free survival (EFS) was calculated from the date of surgery or the first course of neoadjuvante chemotherapy to the date of event (loco regional or metastatic relapse, or death) or last follow up. Patients with stage IV disease at diagnosis were excluded from the statistical evaluation of EFS. Overall survival (OS) was calculated from the date of diagnosis (fin needles aspiration, biopsies or radical mastectomy) to the date of death or last follow-up.

### Statistical analysis

SPSS13.0 software was used for statistical analysis. Descriptive of clinical data were expressed in percentage or median or mean ± SD. Survival was estimated by the Kaplan Meier method, and compared by the log rank test. The relationship between each of the explanatory variables and outcome (EFS and OS) was assessed in turn using univariate and multivariate Cox's regression analysis. A p value of < 0.05 was considered significant.

#### Consent and statement of ethical approval

As the treatment of each patient was decided by the medical staff of the centre, oral consent was obtained from the subjects and was approved by the institutional review boards of the National Institute of Oncology, Cancer Centre in Rabat. This study was approved by the institutional review boards of National Institute of Oncology, in Rabat.

## Results

### Clinical characteristics of all patients

Four hundred and twenty seven patients aged 35 years or younger were diagnosed with breast cancer between January 2003 and December 2007. Four hundred and nine patients were included. The median follow-up time was 32.2 months (range 2- 84.8 months). Table 1 summarizes patient characteristics. The median age at diagnosis was 32 years (range 15-35 years). Twenty-eight patients (6.8%) had a family history of breast cancer. BRCA mutation was sought in only a single case and was present. One hundred and twenty one patients (59%) had nursing antecedents and 129 patients (31.5%) reported use of oral contraceptives. Three hundred and sixty patients (88.2%) had infiltrating ductal carcinoma. Two hundred and six cases (50.4%) were grade II Scarff-Bloom-Richardson (SBR), 53.8% of the cases were estrogen receptors positive, and 61.6% of the cases were progesterone receptors positive. The Her2 status gene amplification was performed in 57 patients. HER-2 was positive in only 28 patients. Fifty three patients (13%) had metastatic disease (stage IV) at first diagnosis, 30 patients (7.3%) had stage I, 140 patients (34.2%) had stage II and the remaining 186 patients (45.5%) had stage III.

**Table 1:** Clinical characteristics of all Patients

Characteristics	No. of patients	(%)
**Nursing**	121	59

**Family history of breast carcinoma**		

Yes	28	6.8

No	254	62.1

Unknown	127	31.1

**SBR grading**		

I	17	4.1

II	206	50.4

III	155	37.9

Unknown	31	7.6

**Estrogen receptor**		

positive	220	53.8

negative	160	39.1

Unknown	29	7.1

**Progesterone receptor**		

Positive	252	61.6

negative	128	31.3

Unknown	29	7.1

**M stage**		

M0	356	87

M1	53	13

**TNM Stage**		

Stage I	30	7.3

Stage II	140	34.2

Stage III	186	45.5

Stage IV	53	13

**HER2**		

Positive	28	6.8

negative	29	7.1

unknown	352	86.1

### Treatment and outcome

#### All patients

**Figure 1:**
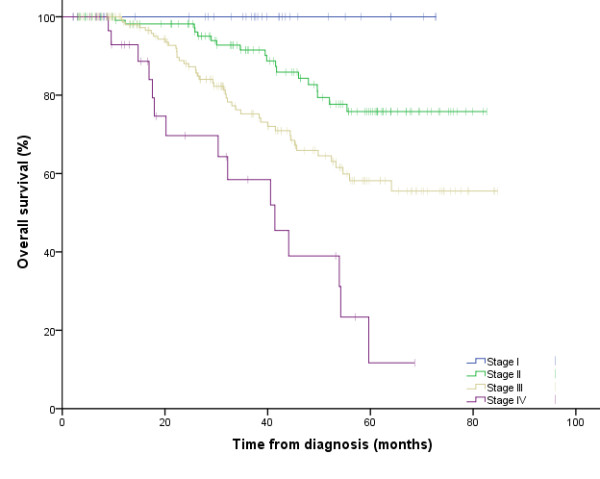
**Survival for all patients according to TNM stage**.

Three hundred and fifty six patients (87%) had local disease at diagnosis and 53 patients (13%) had metastatic disease. At the end of the study period, 92 patients (22.49%) died. For all patients, survival at 3 years was 80.6%. Overall survival rate at 3 years was 100%, 89.3%, 74.7%, and 57.8% in stage I, stage II, stage III and stage IV respectively. This difference was statistically significant: Log rank test, p < 0.001 (figure 1).

#### Metastatic disease

Among 53 patients with metastatic disease, 18 patients (34%) received surgical treatment. Forty seven patients (88.7%) received anthracycline-based regimen in the first line metastatic chemotherapy: thirty-seven patients (78.7%) received AC 60 protocol, six patients (12.8%) received FEC100 protocol and four patients (8.5%) received AT protocol. Trastuzumab was not prescribed for any patients with Her 2 positive status (Table 2).

**Table 2:** Treatment characteristics and overall survival in patients with metastatic disease

	No. of patients(%)	3-Year OS(%)	Median OS		p
				
			Months	95%CI	
**Surgery**					

Yes	18(34)	-	-	-	-
	
No	35 (66)	-	-	-	

**Metastatic First line Chemotherapy**					

No chemotherapy	6(11.3)	50	20.2	0 - 40.4	0.40
	
Chemotherapy	47(88.7)	57.7	39	30.6 - 47.3	

**Chemotherapy protocol**					

AC 60	37(78.7)	57.7	41.4	21.6 - 61.2	0.65
	
FEC 100	6(12.8)	33.3	35.1	30.4 - 39.8	
	
AT	4(8.5)	NR	39	NR	

**Hormonotherapy**					
	
No hormonotherapy	41(77.4)	58.3	39	27.1 -50.8	0.60

hormonotherapy	12(22.6)	48.4	35.1	5.5 - 64.8	

No significant difference in OS at 3 years was seen between the 2 groups of patients how received chemotherapy or not received chemotherapy (57.7% vs 50% respectively; Log rank test: p = 0.4) (Table 2).

#### Non metastatic disease

From three hundred and fifty six patients how had localized disease, 186 patients (52.2%) had stage III at diagnosis, 140 patients (39.3%) with stage II and 30 patients (8.4%) with stage I (table 3). Positive estrogen receptors status was significantly associated with OS and EFS at 3 years compared to negative estrogen receptors status (p = 0.001). Three hundred and forty seven patients (97.5%) received surgery in which 80 patients (23.2%) had conservative surgery (tumorectomy with axillary lymph nodes). The remaining patients 76.8% received radical mastectomy with axillary lymph nodes dissection (Patey type mastectomy) (table 3). All patients with local disease, who were operated, received optimal surgery with free histological margins. Patients with advanced tumors or inflammatory breast cancer have received neoadjuvante chemotherapy before surgery. No significant difference in OS and EFS at 3 years was seen in patients how had radical or conserving surgery.(Table 4).

**Table 3:** Treatments and Disease characteristics in patients with local disease

Characteristic	No. of patients	(%)
**No. of patients**	**356**	**100**

**Tumor size**		

T1	48	13.5

T2	161	45.2

T3	71	19.9

T4	76	21.3

**N Statut**		

N0	107	30.1

N1	74	20.8

N2	93	26.1

N3	82	23

**HER 2**		

HER 2 negative	23	48.9

HER 2 positive	24	51.1

**TNM**		

Stage I	30	8.4

Stage II	140	39.3

Stage III	186	52.2

**Surgery**		

Yes	347	97.5

No	9	2.5

**Surgery type**		

Radical mastectomy	265	76.8

Concerving surgery	80	23.2

**Radiotherapy**		

Yes	290	81.5

No	66	18.5

**Neoadjuvante chemotherapy**		

Yes	76	21.3

No	280	78.7

**Neoadjuvante chemotherapy type**		

Anthracycline	69	90.8

CMF	5	6.6

AC60/TAXANE	2	2.6

**Histological response to neoadjuvante chemotherapy**		

Chevalier 1	2	2.6

Chevalier 2	6	7.9

Chevalier 3	38	50

Chevalier 4	30	39.5

**Adjuvant chemotherapy**		

Yes	311	87.4

No	45	12.6

**Adjuvant hormonotherapy**		

Yes	232	64.6

No	124	35.4

**Table 4:** OS and EFS in patients35 years and younger with local breast cancer

	No. of patients	3-Year OS(%)	P	3-Year EFS(%)	P
Stage I-III	356	83.2	-	62.5	-
**Tumor size**					

T1	48	89.3	0.76	75.9	0.32
			
T2	161	85.5		65	
			
T3	71	79.5		56	
			
T4	76	77.5		55.2	

**SBR grading**					

SBR I	14	100	0.20	67.5	0.45
			
SBR II	176	84.9		63.2	
			
SBR III	137	78		58.8	

**ER**					

ER positive	179	94.4	0.001	75.8	0.001
			
ER negative	149	70.2		50.6	

**N stage**					

N0	107	79.4	0.32	59.8	0.20
			
N+	249	84.8		63.6	

**Surgery type**					

Radical mastectomy	265	82.3	0.17	61.9	0.059
			
Conserving surgery	80	87.7		70.4	

**Adjuvant radiotherapy**					

Yes	290	85.2	0.002	65.7	0.001
			
No	66	70.2		48.6	

**Adjuvant chemotherapy**					

No	45	65.4	0.04	44.7	0.001
			
Yes	311	85		64.9	

**Hormonotherapy**					

Yes	232	85.9	0.001	69.5	0.001
			
No	124	76.6		48	

**Stage**					

I	30	100	0.001	93	0.001
			
II	140	89.4		76.3	
			
III	186	74.7		46.8	

ER. estrogen receptors; N. lymph node; CI. confidence interval; OS. overall survival; EFS. event free survival.

Seventy six patients (21.3%) received neoadjuvante chemotherapy wich 69 patients (90.8%) had Anthracycline based chemotherapy (AC60, FAC50 and FEC100), five patients (6.6%) received CMF protocol and two patients (2.6%) had Anthracycline and taxane protocol (3 cycles of AC60 folowed by 3 cycles of docetaxel). Only two patients had complete response to neoadjuvante chemotherapy. Most patients had a chevalier response 3 and 4 (50 and 39.5% respectively) (table 3).

From 311 patients how received adjuvant chemotherapy, 273 patients (87.8%) had Anthracycline based chemotherapy (AC60, FAC50 and FEC100), 13 patients (4.2%) had sequential Anthracycline and docetaxel, 14 patients (4.5%) received CMF protocol and 11 patients (3.5%) had docetaxel as adjuvant chemotherapy (table 3). Fifty five patients received previous neoadjuvante chemotherapy.

OS and EFS at 3 years were higher in patients how received adjuvant chemotherapy. (Table 5) Twenty-four patients had HER 2 positive and none of them has received trastuzumab (table 3).

**Table 5:** Cox Proportional Hazards Model of Overall Survival for patients with local disease:

	Univariate analysis			Multivariate analysis		
				
	Hazard ratio	95% CI	*P*-value	Hazard ratio	95% CI	*P*-value
**Family history**						

No	1.00			1.00		

Yes	1.10	0.47 - 2.58	0.83	1.12	0.44-2.88	0.81

**SBR grading**						

SBR I	1.00			1.00		

SBR II	2.01	0.27 - 14.6	0.49	1.26	0.15-10.9	0.83

SBR III	2.55	0.35 - 18.69	0.36	1.03	0.12-8.76	0.97

**Tumor size**						

T1	1.00			1.00		

T2	1.54	0.65 - 3.66	0.33	2.25	0.50-10.19	0.30

T3	1.55	0.59 - 4.1	0.38	2.50	0.47-13.42	0.29

T4	1.64	0.64 - 4.23	0. 31	2.34	0.44-12.44	0.32

**N statut**						

N-	1.00			1.00		

N+	0.78	0.47 - 1.28	0.32	0.62	0.29-1.30	0.20

**Stage**						

I	1.00			1.00		

II	2.10	0.53 - 8.37	0.29	1.43	0.24-8.57	0.69

III	4.24	1.1 - 16.28	0.03	3.56	0.65-19.57	0.14

**ER**						

ER positive	1.00			1.00		

ER negative	2.25	1.37 - 3.72	0.001	2.54	1.32- 4.90	0.005

**Adjuvant chemotherapy**						

No	1.00			1.00		

Yes	0.49	0.24 - 0.99	0.04	0.74	0.18-3.05	0.68

**Adjuvant hormonotherapy**						

No	1.00			1.00		

Yes	0.45	0.28 - 0.73	0.001	0.89	0.45-1.77	0.74

**Surgery type**						

Radical mastectomy	1.00			1.00		

Conserving surgery	0.64	0. 34 - 1.23	0.17	0.50	0.19-1.18	0.11

**Adjuvant radiotherapy**						

No	1.00			1.00		

Yes	0.42	0.24-0.74	0.003	0.74	0.25-2.23	0.60

1. Reference; ER. estrogen receptors; N. lymph node; CI. confidence interval; OS. overall survival; EFS. Events free survival.

According to standard recommendations for localised breast cancer when lymph nodes were affected, 290 patients (81.5%) received adjuvant radiotherapy (table3). OS and EFS at 3 years were higher in patients how received adjuvant radiotherapy compared to patients how not received adjuvant radiotherapy. The difference was statistically significant (table 4). From 232 patients how had hormone therapy, 230 patients (64.6%) received tamoxifen in adjuvant setting in the case of positive hormone receptors status and two patients received castration (table 3). OS and EFS at 3 years was statistically significant for patients how received hormone therapy (85.9 vs 76.6%; p = 0,001 and 69.5 vs 48%; p = 0,001 respectively).

At last follow up, forty four patients (12.4%) experienced local relapse, ninety two patients (25.8%) had metastatic progression and sixty nine patients (19.4%) died. For all patients with localised disease, OS and EFS at 3 years were 83.2 and 62.5% respectively.

#### Univariate and multivariate Cox regression analysis

##### Factors influencing survival

Univariate analysis showed that the factors significantly influencing OS were the negative ER status, advanced tumoral stage, absence of adjuvant chemotherapy and adjuvant endocrine therapy for patients with positive ER, and absence of adjuvant radiotherapy (table 5). Multivariate analysis showed that only negative ER was significantly associated with poorer overall survival. (HR = 2.54, 95% CI = 1.32- 4.90, p < 0.005) (table 5)

##### Factors influencing event free survival

Univariate analysis indicated that advanced tumoral stage, negative ER, absence of adjuvant chemotherapy, radiotherapy, and hormone therapy, and conservative surgery were all risk factors of poorer event free survival (table 6), however multivariate analysis showed that negative ER status and advanced stage III disease were the only factors associated with poorer event free survival (table 6).

**Table 6:** Cox Proportional Hazards Model of Event free survival for patients with local disease

	Univariate analysis			Multivariate analysis		
				
	Hazard ratio	95% CI	*P*-value	Hazard ratio	95% CI	*P*-value
**Family history**						

No	1.00			1.00		

Yes	1.25	0.66 - 2.34	0.49	0.86	0.41-1.84	0.71

**SBR grading**						

SBR I	1.00			1.00		

SBR II	0.66	0.26 - 1.64	0.36	0.62	0.17-2.25	0.47

SBR III	0.79	0.32 - 1.99	0.63	0.69	1.19-2.47	0.57

**Tumor size**						

T1	1.00			1.00		

T2	1.26	0.69 - 2.30	0.45	1.27	0.51-3.17	0.61

T3	1.68	0.87 - 3.23	0.12	1.10	0.35-3.14	0.93

T4	1.56	0.82 - 2.98	0.17	1.40	0.48-3.81	0.57

**N statut**						

N-	1.00			1.00		

N+	0.79	0.55 - 1.13	0.20	0.74	0.42-1.30	0.29

**Stage**						

I	1.00			1.00		

II	4.33	1.04 - 17.9	0.04	2.03	0.57 - 7.25	0.27

III	10.96	2.70 - 44.5	0.01	5.35	1.60-17.84	0.01

**ER**						

ER positive	1.00			1.00		

ER negative	1.83	1.26 - 2.64	0.01	1.63	1.00 - 2.67	0.05

**Adjuvant chemotherapy**						

No	1.00			1.00		

Yes	0.45	0.28 - 0.73	0.001	0.90	0.33 -2.52	0.85

**Adjuvant hormonotherapy**						

No	1.00			1.00		

Yes	0.43	0.30 - 0.60	0.001	0.73	0.43 -1.23	0.24

**Surgery type**						

Radical mastectomy	1.00			1.00		

Conserving surgery	0.63	0.40 - 1.01	0.05	0.74	0.42 -1.13	0.30

**Adjuvant radiotherapy**						

No	1.00			1.00		

Yes	0.45	0.30 - 0.68	0.001	0.82	0.35-1.19	0.64

## Discussion

This work conducted at the national institute of oncology in morocco analyzed the epidemiological, clinical, therapeutic and prognostic characteristics of breast cancer in women aged 35 years or less and the relationship between these characteristics and outcome (EFS and OS).

Of 5309 patients diagnosed with breast cancer between 2003 and 2007, 8% of the cases were aged 35 years or younger. The higher age adjusted incidence could be partially attributed to the higher proportion of young women in the general population. In the US, the women aged 35 years or younger represent only 2.7% of new cases [10]. In Asian series, this number varies between 10% in developed and up to 24% in developing countries [11].

This study found less than 10% of family history of breast cancer and no evidence for a relationship between family history of breast cancer and survival. However, family history of breast cancer is an important indicator of risk in young women. In a Swedish population-based study of 262 women with breast cancer aged 40 years or younger, 48% of patients had a family history of breast or ovarian cancer [12].

The reports studied the impact of age and others prognostic factors showed that high tumor grade represents an individual prognostic factor in younger and older premenopausal patients [13]. In addition, several studies published after 1995 reported that the unfavorable impact of young age on survival was present only in patients who did not receive chemotherapy [14]. However, Colleoni et al. [15], and Kothari et al. [16], showed that the outcome of patients aged ≤ 35 years was less favorable than those aged > 35 years. In fact, the outcome of young women with localized disease included in the present study is less favorable than that of patients having localized disease followed in the same Institute and included in a previous study [17]. Also, in this work, we found that women were more likely to receive adjuvant chemotherapy and they had a better survival, however high tumor grade was not influenced survival.

Our findings revealed that negative ER status was associated with lower survival and event free survival in univariate and multivariate analysis. Curigliano [18] and Aebi [19] noted that the positive ER status in young patients seems to have a different prognostic value. Data suggest that very young women with endocrine-responsive tumors had a statistically significant higher risk of disease recurrence than older premenopausal patients. In contrast, results in younger and older premenopausal patients were similar in the case of the ER positive status. However, Ana M. Gonzalez-Angulo[20] et all found that Hormone receptor negativity was associated with shorter RFS and OS. This result was consistent with our finding.

Breast cancer in young women is frequently diagnosed at advanced stage [21]. Consequently, the majority of patients received radical mastectomy. In addition, a positive family history or the presence of a BRCA1/2 gene mutation will also influence decisions for mastectomy rather than breast conservation [3]. Until now, there is still no final conclusion about whether age is a risk factor of local recurrence in breast conserved cases, and there are still inconsistent findings in previous clinical studies. But it was reported in most studies that among those who received breast conserving surgery, young patients had a higher local recurrence rate. [22-28] Over 20% of our patients with local disease received conservative surgery. These patients had lower event-free survival than patients who received radical mastectomy. The difference tended towards significance.

The updated Early Breast Cancer Clinical Trialists' meta-analysis have shown beneficial effects of adjuvant tamoxifen in younger women [29], as well as the findings from the International Breast Cancer Study Group (Trial 13-93) [30]. In our series, the patients with localised disease and estrogen receptor positive status, how had received the hormonal treatment had better OS and EFS.

However our retrospective study showed several limitations because it implicated potential bias in the choice of treatment. In addition, the lake of cytogenetic investigation of BRCA gene mutation due to the low socio-economic level of these patients and the lake of Her-2 gene amplification test in the majority of our patients are 2 major limitations.

## Conclusion

In Morocco, the incidence of breast cancer in young women aged ≤ 35 is higher compared than that in developed countries. In this subgroup of patients, the invasive breast cancer has more aggressive behaviors. In addition, the ER negative status was associated with lower EFS and OS. Further research program and clinical trials were needed in young Moroccan breast cancer women improve their management and their outcome.

## List of abriviations

**CI: **Confidence interval; **ER: **Estrogen receptor; **EFS: **Event free survival; **HR: **Hazard ratio; **IHC: **Immunohistochemistry; **N: **lymph nodes; **OS: **Overall surviaval; **SD: **Standard deviation.

## Competing interests

The authors declare that they have no competing interests.

## Authors' contributions

**HA **has conceived the study, exploited data, coordinated, drafted and wrote the manuscript. **IL**, **FEM **and **NI **participated in the design and data exploitation and its input. **RA, NI, RR **and **MAT **have performed the statistical analysis. **RA, NI **and **MAT **revised the manuscript. **HM, OEM, NB, RA **and **HE **participated in the design of this study, and revised the manuscript. All authors read and approved the final manuscript.
